# Variable levels of spike and ORF1ab RNA in post-mortem lung samples of SARS-CoV-2-positive subjects: comparison between ISH and RT-PCR

**DOI:** 10.1007/s00428-021-03262-8

**Published:** 2022-02-01

**Authors:** Federica Zito Marino, Tiziana De Cristofaro, Massimo Varriale, Giuseppa Zannini, Andrea Ronchi, Elvira La Mantia, Carlo Pietro Campobasso, Francesco De Micco, Pasquale Mascolo, Maurizio Municinò, Emilia Municinò, Francesco Vestini, Omero Pinto, Marta Moccia, Noè De Stefano, Oscar Nappi, Carmen Sementa, Giovanni Zotti, Lamberto Pianese, Carmela Giordano, Renato Franco

**Affiliations:** 1 Pathology Unit, Department of Mental and Physical Health and Preventive Medicine, Università degli Studi della Campania “L Vanvitelli”, via Luciano Armanni 5, 80138 Naples, Italy; 2 IEOS - Institute of Experimental Endocrinology and Oncology ‘G. Salvatore’, National Research Council, via S. Pansini 5, 80131 Naples, Italy; 3 BioMol Laboratories srl, Corso San Giovanni 849, 80146 Naples, Italy; 4 Department of Experimental Medicine, University of Campania, Luigi Vanvitelli, Naples, Italy; 5 Forensic Medicine Unit, “S. Giuliano” Hospital, Giugliano in Campania, Italy; 6 Pathology Unit, “San Giuseppe Moscati” Hospital, Avellino, Italy; 7 Istituto Diagnostico “Varelli”, Naples, Italy; 8 Forensic Medicine Unit, AORN “San Giuseppe Moscati, Contrada Amoretta, 83100 Avellino, Italy; 9 Forensic Medicine Unit, ASL Salerno, Salerno, Italy; 10 Forensic Medicine Unit, ASL Avellino, Avellino, Italy; 11 Forensic Medicine Unit, UOPC ASL NA3sud, Naples, Italy

**Keywords:** SARS-CoV-2, Reverse transcription polymerase chain reaction (RT-PCR), In situ hybridization (ISH), Post-mortem lung samples, Spike(S), Open reading frame (ORF1ab)

## Abstract

Post-mortem examination plays a pivotal role in understanding the pathobiology of the SARS-CoV-2; thus, the optimization of virus detection on the post-mortem formalin-fixed paraffin-embedded (FFPE) tissue is needed. Different techniques are available for the identification of the SARS-CoV-2, including reverse transcription polymerase chain reaction (RT-PCR), immunohistochemistry (IHC), in situ hybridization (ISH), and electron microscopy. The main goal of this study is to compare ISH versus RT-PCR to detect SARS-CoV-2 on post-mortem lung samples of positive deceased subjects. A total of 27 samples were analyzed by RT-PCR targeting different viral RNA sequences of SARS-CoV-2, including envelope (E), nucleocapsid (N), spike (S), and open reading frame (ORF1ab) genes and ISH targeting S and Orf1ab. All 27 cases showed the N gene amplification, 22 out of 27 the E gene amplification, 26 out of 27 the S gene amplification, and only 6 the ORF1ab gene amplification. The S ISH was positive only in 12 out of 26 cases positive by RT-PCR. The S ISH positive cases with strong and diffuse staining showed a correlation with low values of the number of the amplification cycles by S RT-PCR suggesting that ISH is a sensitive assay mainly in cases carrying high levels of S RNA. In conclusion, our findings demonstrated that ISH assay has lower sensitivity to detect SARS-CoV-2 in FFPE compared to RT-PCR; however, it is able to localize the virus in the cellular context since it preserves the morphology.

**Supplementary Information:**

The online version contains supplementary material available at 10.1007/s00428-021-03262-8.

## Introduction

In January 2020, a severe acute respiratory syndrome, caused by the Novel Coronavirus 2019 SARS-CoV-2, was firstly identified in China and quickly spread throughout the world. The World Health Organization (WHO) has officially declared the SARS-CoV-2 disease a pandemic with a public health emergency of international concern. SARS-CoV-2 belongs to the family of the coronaviruses (CoVs) that are enveloped, positive-sense, single-stranded RNA viruses. Before the infection caused by SARS-CoV-2, SARS-CoV and Middle East respiratory syndrome coronavirus (MERS-CoV) are the best-known examples of large-scale epidemic coronavirus-associated involvement in severe acute respiratory syndromes [1].

Phylogenetically, the SARS-CoV-2 genome is closely related to two bat coronaviruses, bat-SLCoVZC45 and bat-SL-CoVZXC21 (89–96.3% sequence homology), while it has less sequence similarity (79–82%) with SARS-CoV and MERS-CoV [2, 3]. The viral structures and genome of SARS-CoV-2 showed a unique feature compared to all other coronaviruses [1].

The genome of SARS-CoV-2 is larger compared to other RNA viruses and it is organized as follows, 5′ to 3′: two flanking untranslated regions (UTRs), a single long open reading frame (ORF1ab), a non-structural polyprotein, four structural proteins—spike (S), envelope (E), membrane (M), nucleocapsid (N)—and five accessory proteins—ORF3a, ORF6, ORF7a, ORF7b, ORF8, and ORF10 [4–6].

The replicase gene ORF1ab encodes a large polyprotein (pp1ab), which is proteolytically cleaved into 16 non-structural proteins (NSPs) that are involved in the transcription and replication of the virus [7]. The N protein forms the helical capsid to accommodate the genome, the M and E are needed for the virus assembly, and the S protein mediates the host cell recognition and the entry of the virus [8]. The S protein is composed of two subunits, the S1 domain responsible for the receptor-binding and the S2 domain associated with the envelope. The S1 domain of SARS-CoV-2 showed only 40% of homology with other coronaviruses and great variability in amino acids resulting in a high affinity for binding to the human receptor angiotensin-converting enzyme 2 (ACE-2) [1]. SARS-CoV-2 shares with other SARS-CoV more than 90% amino acid identity of the structural proteins, excluding the S gene, which diverges [3, 9, 10].

The SARS-CoV-2 diagnosis is currently based on the clinical manifestations associated with the detection of virus RNA through real-time reverse transcription polymerase chain reaction (RT-PCR), primarily in the nasopharyngeal and oropharyngeal swabs [11].

To date, the RT-PCR is the gold standard to identifying the SARS-CoV-2 infection according to the WHO recommendations [11].

The RT-PCR assays currently available target E, N, S, and ORF1b (including RNA-dependent RNA polymerase-RdRp) genes [1, 10, 12].

The rapid spread and the limited knowledge of the virus require continuous updating by the experts improving particularly the investigation on post-mortem biomaterials. The post-mortem analysis represents a pivotal tool in understanding the biological characteristics and the pathogenesis of SARS-CoV-2.

The SARS-CoV-2 RT-PCR is currently standardized exclusively in fresh samples, while the detection of the virus on the post-mortem formalin-fixed paraffin-embedded (FFPE) tissue represents an open issue. Since the autopsy material can be very heterogeneous and subject to numerous pre-analytical limitations, the optimization of the methods to identify the virus on FFPE is required.

The identification of SARS-CoV-2 infection on FFPE specimens may be carried out by different methods including RT-PCR, immunohistochemistry (IHC), in situ hybridization (ISH), and electron microscopy [13–16]. Previous studies showed the use of IHC in identifying SARS-CoV-2 in post-mortem biomaterial reporting a sensitivity of 85.7% and specificity of 53.3% compared to RT-PCR. The RNA ISH test could represent a very useful technique to characterize SARS-CoV-2 on the FFPE tissue, since this method leads to virus detection preserving the morphological features. To date, few data have been reported about the use of ISH assay for detection of viral RNA in autopsies [17–19]. The RNAscope technology, currently commercially available, proposed two different probes one targeting the SARS-CoV-2 spike protein and the other targeting the Orf1ab sense RNA strand produced during viral replication. The electron microscopy based on the identification of SARS-CoV-2 virus particles in the ultrastructural morphology context represents a not feasible detection method in routine clinical practice; thus, it is not performed other than for research use only.

To date, few data have been reported about the comparison between molecular methods to the SARS-CoV-2 RNA detection on post-mortem samples, particularly regarding ISH assay versus RT-PCR. The main aim of this study is the comparison between ISH and RT-PCR to detect RNA SARS-CoV-2 on post-mortem lung samples from positive patients, in order to assess the sensibility and the specificity of these assays.

The present study shows the SARS-CoV-2 RNA analysis on a series of post-mortem lung samples performed by the RT-PCR based on an extensive panel of targets, including E, N, S, and Orf1ab genes, and the ISH method using two different probes, such as the Spike and the Orf1ab sense RNA strand produced.

## Materials and methods

### Cases collection

Autopsy cases of subjects affected by COVID-19 performed between March 2020 and December 2020 in Campania (Italy) were collected. Inclusion criteria were (1) positive molecular nasopharyngeal swab before or at the moment of hospitalization, (2) availability of clinical features, and (3) availability of histological material for all the analysis. The cohort included 15 males and 12 females. The age of the subjects was between 45 and 82 years (mean age: 66.7 years). All patients had some comorbidities, including arterial hypertension (20 out of 27, 74%), cardiac hypertrophy (10 out of 27, 37%), obesity (body mass index >30) (5 out of 27, 18.5%), diabetes mellitus type 2 (4 out of 27, 14.8%), obstructive chronic broncho-pneumopathy (3 out of 27, 11.1%), and chronic nephropathy (2 out of 27, 7.4%). All patients have been hospitalized and time of hospitalization was variable, between 20 and 122 days. Autopsies were performed between 3 and 5 days after the death of the subjects. All clinical features are summarized in Supp Table 1. Consent to perform the autopsy was given in all cases by the Attorney’s Office of Naples.

**Table 1:** Cycle threshold (Ct) by RT-PCR and ISH results for each case of our series

Case	RNase P Ct	RT-PCR Ct				ISH score	
		E	N	S	ORF1ab	S	ORF1ab
1	30.3	–	33.6	38.4	–	0	0
2	29.1	32.2	31.0	35.0	–	0	0
3	31.5	33.4	30.6	35.7	–	0	0
4	36.8	35.5	32.6	38.6	–	0	0
5	35.2	30.6	27.7	33.9	–	0	0
6	26.5	31.1	27.5	33.3	–	0	0
7	34.4	18.8	15.8	23.9	34.8	2+	0
8	31.0	36.9	29.9	36.3	–	0	0
9	28.9	32.3	29.0	34.5	–	0	0
10	27.1	–	30.4	36.6	–	0	0
11	34.2	25.8	22.6	27.9	34.0	1+	NV
12	37.4	30.0	26.1	33.2	–	0	0
13	31.6	29.7	26.8	32.2	29.5	0	0
14	36.1	20.0	16.9	21.0	27.5	2+	NV
15	32.6	21.4	19.2	22.8	29.9	2+	2+
16	30.6	22.5	20.5	24.4	–	1+	0
17	28.2	24.9	22.6	23.9	24.4	1+	2+
18	35.2	36.2	29.8	35.5	–	1+	0
19	30.4	33.4	31.7	37.5	–	1+	0
20	27.7	–	32.1	39.4	–	0	0
21	30.2	35.6	29.3	35.3	–	0	0
22	33.2	28.5	24.3	31.6	–	1+	0
23	34.6	30.3	25.7	33.9	–	1+	0
24	37.0	41.6	30.7	37.2	–	1+	0
25	34.3	23.1	19.5	22.2	–	2+	0
26	35.4	–	31.3	34.3	–	0	0
27	29.3	–	36.3	–	–	0	0

*NV*, not evaluable; *−*, negative

### Histological evaluation

Formalin-fixed and paraffin-embedded (FFPE) tissues from lungs of the 27 subjects were collected. All cases were fixed in formalin for at least 72 h. The tissues were submitted to the standard procedures for histological evaluation. Sections of 4 μm in thickness were cut from each block, and stained by hematoxylin and eosin. Two expert pathologists (RF and AR) evaluated all the histological slides.

### SARS-CoV-2 detection by real-time RT-PCR assays

RNeasy FFPE Kit (QIAGEN, cat. no. 73504) was used to extract SARS-CoV-2 RNAs from FFPE tissues. Extraction was performed according to the manufacturer’s instructions. RNA was eluted with 30 μL buffer and used for RT-PCR assay. Viral 3 SARS-CoV-2 kit (BioMol Laboratories srl, Italy), which targets envelope gene (*E*) of *Sarbecovirus*, nucleocapsid (*N*), and ORF1ab genes of SARS-CoV-2, was used for SARS-CoV-2 RNA detection according to the manufacturer’s instructions. Human RNase P gene was used as housekeeping gene. SARS-CoV-2-positive control is a synthetic RNA transcript containing five gene targets (E, N, ORF1ab, RdRP, and S genes of SARS-CoV-2) and human RNase P gene. Briefly, 10 μL of extracted RNA was added to 5 μL of 4× real-time Mix PCR and 5 μL of Primer-Probes Mix. The CFX-96 real-time thermal cycler (Bio-Rad Laboratories, Inc., Hercules, CA, USA) was used for amplification. The conditions consisted of 1 cycle of 2 min at 25 °C, 15 min at 50 °C, and 3 min at 95 °C, followed by 44 cycles of 3 s at 94 °C and 60 s at 60 °C. Primers (F: TCA ACT CAG GAC TTG TTC TTA CCT and R: TGG TAG GAC AGG GTT ATC AAA C) and probe (6-FAM-TTC CAT GCT ATA CAT GTC TCT GGG A-BHQ-1) (Metabion International AG) were used for Spike (S) gene amplification, using the same thermal PCR profile of Viral 3 SARS-CoV-2 kit.

### In situ hybridization (ISH)

We performed in situ hybridization (ISH) to identify Spike and ORF1ab of SARS-CoV-2 in the pulmonary tissue using RNAscope 2.5 high-definition detection kit (Advanced Cell Diagnostics) and two specific probes: Spike (V-nCoV2019-S probe) and ORF1ab (V-nCoV2019-orf1ab-sense-C2). Sections of 4 μm in thickness from each block were obtained. The sections were deparaffinized in xylene and placed at 95 °C for 10 min. The peroxidase activity was stopped by 10-min hydrogen peroxide incubation followed by permeabilization using protease plus treatment at 40 °C for 30 min. The next 2 h were used for the hybridization of the probe at 40 °C. The signal of the RNAscope was developed with 3,3′-diaminobenzidine and the nuclei were counterstained using the hematoxylin. All samples were also tested for RNAscope® 2.5 LS Positive Control Probe-Hs-PPIB (cat. no. 313908) to confirmed well-preserved RNA. A normal autopsy pulmonary tissue was added to each slide for negative control. We defined a score combining staining intensity and diffusion for the interpretation of both probes, as the following:

- Score 0 (negative): no positive staining
- Score 1+ (focally positive): slight and occasional staining
- Score 2+ (diffusely positive): moderate to strong, diffuse staining

The samples that showed indistinguishable dot signals, hemosiderin deposits, and endogenous pigments have been defined as not evaluable. The evaluation of ISH test was carried out by two different blinded observers (RF and AR).

## Results

### SARS-CoV-2 RT-PCR

Viral 3 SARS-CoV-2 kit (BioMol Laboratories srl) uses the expression of human RNase P gene (NM-) as endogen control. The amplification of the RNase P gene was satisfactory in 26 out of 27 post-mortem lung samples analyzed. Although in case 12 the amplification of the RNase P gene was not satisfactory, however, the amplification of the E, N, and S genes was detected demonstrating the presence of the virus (Table 1).

**Fig. 1:**
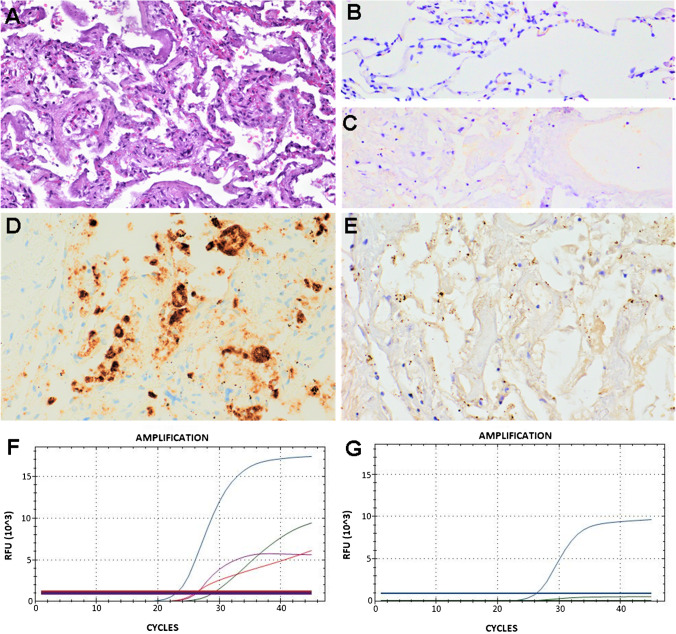
Representative results of case 15. **A** Hematoxylin and eosin staining (original magnification 400×); **B** negative control of normal lung tissue (original magnification 400×); **C** negative control of non-COVID-19 ARDS lung tissue (original magnification 400×); **D** S SARS-CoV-2 ISH positive staining score 2+ (original magnification 400x); **E** ORF1ab ISH positive staining score 2+ (original magnification 400×); **F** N viral gene (blue line), E viral gene (violet line), ORF1ab viral gene (red line), and RNase P human gene (green line) amplification by RT-PCR; **G** S viral gene (blue line) and D69–70 S viral gene (green line) amplification by RT-PCR. The horizontal lines, parallel to *x*-axis, represent the single threshold line (FAM, HEX, Texas-Red, and Cy5)

**Fig. 2:**
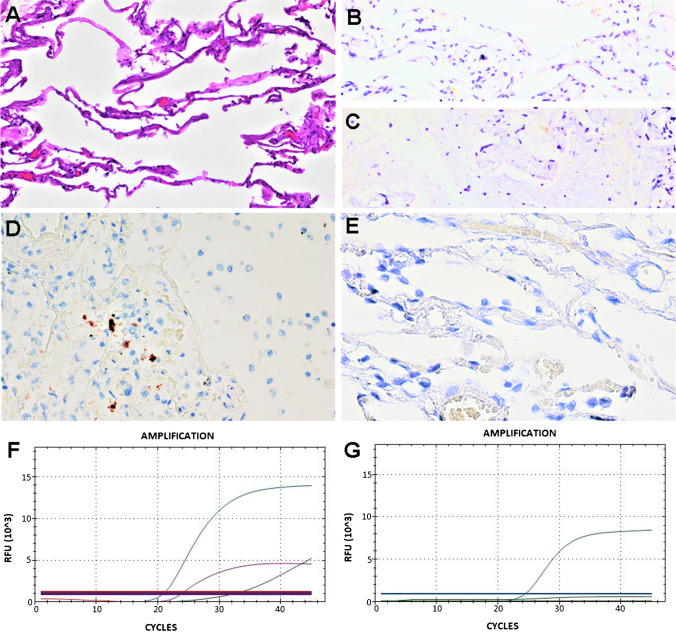
Representative results of case 16. **A** Hematoxylin and eosin staining (original magnification 400×); **B** negative control of normal lung tissue (original magnification 400×); **C** negative control of non-COVID-19 ARDS lung tissue (original magnification 400×); **D** S SARS-CoV-2 ISH positive staining score 1+ (original magnification 400×); **E** ORF1ab ISH negative (original magnification 400×); **F** N viral gene (blue line), E viral gene (violet line), ORF1ab viral gene (red line), and RNase P human gene (green line) amplification by RT-PCR; **G** S viral gene (blue line) and D69–70 S viral gene (green line) amplification by RT-PCR. The horizontal lines, parallel to *x*-axis, represent the single threshold line (FAM, HEX, Texas-Red, and Cy5)

All 27 cases showed the N gene amplification, while only 22 out of 27 (81.5%) cases showed also the E gene amplification. The S gene amplification was detected in 26 out of 27 (96.3%) cases analyzed (Figs. 1 and 2). Among the 27 cases analyzed, only 6 (22.2%) cases showed the ORF1ab gene amplification demonstrating the presence of the virus in the active replication phase (Table 2 and Figs. 1 and 2).

**Table 2:** Comparison of S/ORF1ab SARS-CoV-2 ISH and RT-PCR targeting E, N, S, and ORF1ab results

SARS-CoV-2 ISH N.27			RT-PCR N.27							
			E		N		S		ORF1ab	
			**+**	**–**	**+**	**–**	**+**	**–**	**+**	**–**
			22 (81.5%)	5 (18.5%)	27 (100%)	0	26 (96.3%)	1 (3.7%)	6 (22.2%)	21 (77.8%)
S	**+**	12 (44.4%)	12 (44.4%)	0	12 (44.4%)	0	12 (44.4%)	0	5 (18.5%)	7 (25.9%)
	**–**	15 (55.6%)	10 (37%)	5 (18.5%)	15 (55.6%)	0	14 (51.9%)	1 (3.7%)	1 (3.7%)	14 (51.9%)
ORF1ab	**+**	2 (7.4%)	2 (7.4%)	0	2 (7.4%)	0	2 (7.4%)	0	2 (7.4%)	0
	**–**	23 (85.2%)	18 (66.7%)	5(18.5%)	23 (85.2%)	0	22 (81.5%)	1 (3.7%)	2 (7.4%)	21 (77.8%)
	**NV**	2 (7.4%)	2 (7.4%)	0	2 (7.4%)	0	2 (7.4%)	0	2 (7.4%)	0

*NV*, not evaluable; *+*, positive; *−*, negative

### SARS-CoV-2 ISH assays

The ISH test using S SARS-CoV-2 probe was positive in 12 out of 27 (44.4%) cases, particularly 8 cases showed slight and occasional staining (score 1+) and 4 cases showed strong and diffuse staining (score 2+) (Table 2 and Figs. 1 and 2).

**Fig. 3:**
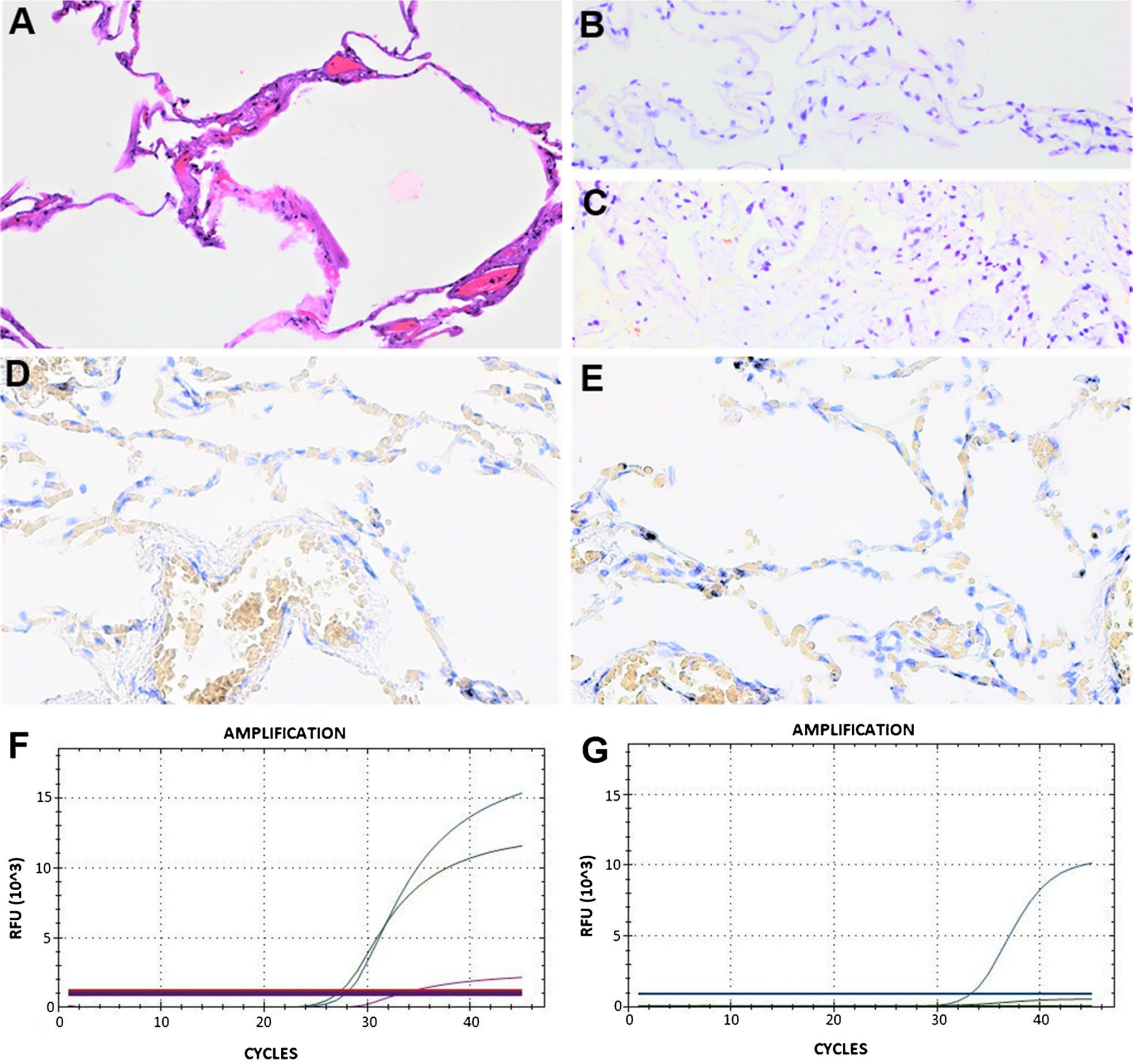
Representative results of case 6. **A** Hematoxylin and eosin staining (original magnification 400×); **B** negative control of normal lung tissue (original magnification 400×); **C** negative control of non-COVID-19 ARDS lung tissue (original magnification 400×); **D** S SARS-CoV-2 ISH negative (original magnification 400×); **E** ORF1ab ISH negative (original magnification 400×); **F** N viral gene (blue line), E viral gene (violet line), ORF1ab viral gene (red line), and RNase P human gene (green line) amplification by RT-PCR; **G** S viral gene (blue line) and D69–70 S viral gene (green line) amplification by RT-PCR. The horizontal lines, parallel to *x*-axis, represent the single threshold line (FAM, HEX, Texas-Red, and Cy5)

The ISH test using ORF1ab SARS-CoV-2 probe was positive only in 2 out of 27 (7.4%), negative in 23 cases out of 27 (85.2%), and not evaluable in 2 cases (7.4%) (Table 2 and Figs. 1, 2, and 3).

The two cases (cases 17 and 15) ORF1ab ISH positive were simultaneously positive for S ISH test (Table 3 and Fig. 1). Both S and ORF1ab ISH staining were generally localized in alveolar macrophages, in air spaces, in hyaline membranes, and in pneumocytes (Table 3).

**Table 3:** Characteristics of S SARS-CoV-2 ISH positive cases

Case	S ISH positive		ORF1ab ISH		RT-PCR			
	Score	Virus localization	Score	Virus localization	E	N	S	ORF1ab
7	2+	Alveolar macrophages, pneumocytes	0		+ (18.8)	+ (15.8)	+ (23.9)	+ (34.8)
14	2+	Alveolar macrophages, air spaces, pneumocytes	NV		+ (20.0)	+ (16.9)	+ (21.0)	+ (27.5)
15	2+	Alveolar macrophages, air spaces, pneumocytes	2+	Alveolar macrophages, pneumocytes	+ (21.4)	+ (19.2)	+ (22.8)	+ (29.9)
25	2+	Alveolar macrophages, air spaces, hyaline membranes, pneumocytes	0		+ (23.1)	+ (19.5)	+ (22.2)	–
11	1+	Air spaces, pneumocytes	NV		+ (25.8)	+ (22.6)	+ (27.9)	+ (34.0)
16	1+	Alveolar macrophages, air spaces, hyaline membranes, pneumocytes	0		+ (22.5)	+ (20.5)	+ (24.4)	–
17	1+	Alveolar macrophages, air spaces, pneumocytes	2+	Alveolar macrophages, pneumocytes	+ (24.9)	+ (22.6)	+ (23.9)	+ (24.4)
18	1+	Alveolar macrophages, air spaces, pneumocytes	0		+ (36.2)	+ (29.8)	+ (35.5)	–
19	1+	Alveolar macrophages, air spaces, pneumocytes	0		+ (33.4)	+ (31.7)	+ (37.5)	–
22	1+	Alveolar macrophages, air spaces, hyaline membranes, pneumocytes	0		+ (28.5)	+ (24.3)	+ (31.6)	–
23	1+	Alveolar macrophages, pneumocytes	0		+ (30.3)	+ (25.7)	+ (33.9)	–
24	1+	Air spaces, pneumocytes	0		+ (41.6)	+ (30.7)	+ (37.2)	–

*NV*, not evaluable; +, positive; −, negative; for each case, the cycle threshold (Ct) by RT-PCR was reported in brackets

### Comparison of RT-PCR and ISH results

Spike SARS-CoV-2 ISH showed positive staining only in 12 out of 26 cases positive by S RT-PCR, suggesting a low sensitivity of the ISH assay. Case 27 was negative both by S RT-PCR and S ISH analysis; exclusively, the N gene amplification was observed in this case. Furthermore, no histological damage was found in this lung tissue compared to all the other cases analyzed.

**Fig. 4:**
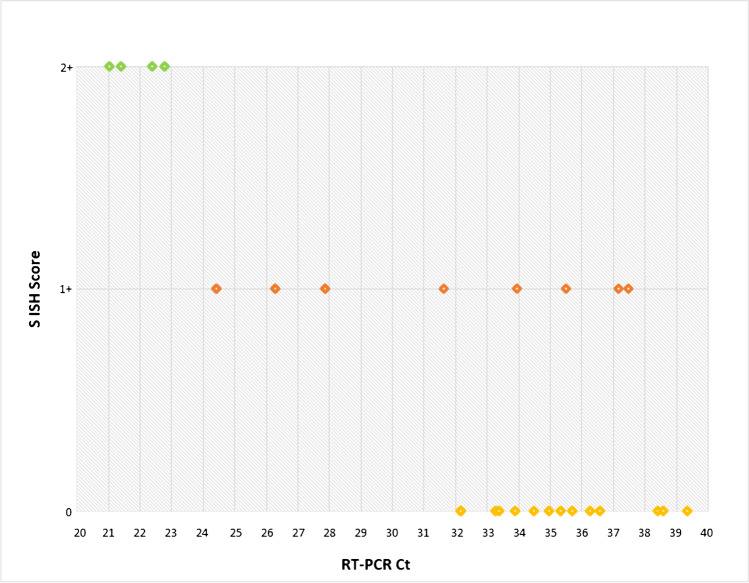
Correlation between S SARS-CoV-2 ISH and the cycle threshold (Ct) values by RT-PCR

The S ISH positive cases with score 2+ showed a lower value of the number of the amplification cycles by S RT-PCR (range 21.0–22.7) compared to those of S ISH positive cases with score 1+ (range 24.4–37.4) and S ISH negative cases (range 32.1–39.3) (Table 3 and Fig. 4).

Among 6 out of the 27 cases ORF1ab positive by RT-PCR, two were also positive by the ORF1ab ISH technique, one was negative, and two not evaluable (Table 2).

## Discussion

The post-mortem biomaterial from SARS-CoV-2-positive deceased subjects constitutes a gold mine for understanding the pathogenesis of the virus, its localization in the different organs and cellular contexts.

In this context, the choice of a sensitive and specific technique is crucial to evaluate the presence of the virus also in extrapulmonary tissues as clarifying the onset of unusual symptoms not known to be associated with the SARS-CoV-2 infection.

Previous studies showed data regarding the virus detection in post-mortem samples of SARS-CoV-2-positive subjects, including both pulmonary and extrapulmonary tissues [18, 20–24] (Table 4).

**Table 4:** Representative results of the main studies currently available regarding the detection of SARS-CoV-2 in post-mortem samples of positive subjects: comparison between ISH and RT-PCR

Ref.	N.Autopsies	Tissue type	RT-PCR results								ISH results					
			E		N		S		ORF1ab		N		S		ORF1ab	
			**+**	**–**	**+**	**–**	**+**	**–**	**+**	**–**	**+**	**–**	**+**	**–**	**+**	**–**
Our data	27	Pulmonary	22	5	27	0	26	1	6	21	NP	NP	12	15	2	23
[18]	8	Pulmonary	6	2	6	2	NP	NP	NP	NP	6	2	6	2	NP	NP
[20]	6	Heart	NP	NP	1	5	1	5	1	5	0	6	NP	NP	NP	NP

+, positive; −, negative; *NP*, not performed

Unfortunately, the detection of the SARS-CoV-2 in the post-mortem biomaterial is limited by several factors, including pre-analytical, analytical, and post-analytical features, but also method-specific bias [17]. The choice of a suitable method for the virus identification on post-mortem FFPE currently represents an urgent challenge for the scientific community.

The RT-PCR is generally the gold standard for the RNA virus diagnosis and also for SARS-CoV-2 detection; the WHO recommends the use of RT-PCR in different kinds of fresh samples such as nasopharyngeal and/or oropharyngeal swabs and lower respiratory specimens (sputum and/or endotracheal aspirate or bronchoalveolar lavage) [19]. To date, no gold standard has yet been defined for SARS-CoV-2 detection on FFPE samples.

Some studies on post-mortem FFPE samples of SARS-CoV-2-positive subjects performed preferentially RT-PCR for the virus identification, using only one structural gene, such as E and N [22, 23]. Other studies performed ISH assay and not RT-PCR to detect SARS-CoV-2 on FFPE samples [21, 24]. Magro and colleagues analyzed 12 autopsies and showed the distribution of SARS-CoV-2 RNA by ISH both in pulmonary and extrapulmonary tissues, including the heart, liver, spleen, and kidney [21]. Ko et al. did not analyzed autopsies; however, they demonstrated the presence of SARS-CoV-2 by ISH on FFPE samples, particularly on the skin biopsies [24].

Discrepant viral evidence results between RT-PCR and ISH in FFPE post-mortem tissue have been the subject of recent studies [18, 20]. Maccio and colleagues analyzed 6 FFPE myocardial tissues showing the presence of SARS-CoV-2 RNA by RT-PCR in 5 out of 6 samples although the viral RNA evidence could not be proven through ISH assay [20].

Massoth and colleagues have reported really interesting data about the detection through RT-PCR and ISH of SARS-CoV-2 in 8 COVID-19 autopsies, including both pulmonary and extrapulmonary samples. The ISH showed a sensitivity of 86.7% and a specificity of 100% compared to RT-PCR assay, but treating tested blocks as separated cases [18].

Our comparison study between ISH and RT-PCR has some novelty points, including Orf1ab ISH analysis and an extensive RT-PCR panel including E, N, S, and Orf1ab. To date, to the best of our knowledge, no data have been reported about Orf1ab detecion; this gene target could play a pivotal role in the evaluation of COVID-19 infection. The WHO recommends for fresh samples the analysis by RT-PCR of at least two structural genes, including E and N, in order to ensure the robustness of the assay [11].

The E gene amplification indicates the presence of the virus, since this gene is highly conserved both in SARS and SARS-CoV-2. Similarly, the N gene, which encodes the nucleocapsid protein specific of the SARS-CoV-2, is used to prove exclusively the presence of the virus. Instead, ORF1ab gene amplification is closely associated with the replicative activity of the virus, rather than only with its presence [25]. The S gene is not usually a target of choice for SARS-CoV-2 diagnosis with RT-PCR, since it is frequently subject to mutations due to high selection pressure; therefore, it is generally used to search possible SARS-CoV-2 mutations [17, 26].

Noteworthy, the analysis of the S gene by RT-PCR played a pivotal role to demonstrate the presence of Spike in post-mortem lung samples, especially in S ISH negative cases. The S ISH showed positive staining only in 44.4% of lung samples analyzed, compared to 96.3% of cases positive by RT-PCR.

In our study, S SARS-CoV-2 ISH assay for detection of virus in post-mortem lung samples showed a sensitivity only of 46% and a specificity of 100% compared to RT-PCR.

Only one lung sample of our series was negative by RT-PCR since it showed exclusively N gene amplification, but it was negative by E, S, and ORF1ab. We evaluated this case as a true negative result by RT-PCR since it was confirmed also by the absence of lung histological damage. On the other hand, all cases with S positive showed significant lung histological damage, as extensive diffuse alveolar damage and lung stroke (*data not shown*).

In our series, the correlation observed between S ISH score 2+ and the lower value of the number of the S gene amplification cycles by RT-PCR suggests that ISH is a sensitive test mainly to detect cases carrying high amounts of Spike RNA. The false-negative cases by ISH could be explained by a reduced amount of S RNA.

Similarly, ORF1ab ISH was positive only in 2 out of 6 cases positive by ORF1ab RT-PCR, suggesting, also in this case, a limit of the ISH to identify ORF1ab positive cases with poor quantity of target RNA.

The RT-PCR targeting E, N, S, and ORF1ab genes has shown high sensitivity and specificity for the identification of the virus on post-mortem samples suggesting its potential use as gold standard also in FFPE samples and not only in fresh samples. The RT-PCR assay used in this study includes also an endogen control, human RNase P gene, that ensured the adequacy and the quality of the extracted RNA showing a high yield despite the difficulty of pre-analytical managing post-mortem materials.

In our series, the RT-PCR results have not been subject to pre-analytical problems generally associated with autopsy biomaterial, including RNA degradation during the post-mortem interval or ischemia time and RNA fragmentation during formalin fixation, described as sources of errors [17].

The major drawback of the RT-PCR is associated with the lack of a possibility to localize the viral RNA in a morphological context in order to define the infection of specific cell types useful for defining the pathogenesis of the SARS-CoV-2-related disease.

In this context, despite a not too high sensitivity, the RNA ISH approach on the post-mortem material from SARS-CoV-2-positive patients could provide new insights improving the characterization of the virus, the development of specific treatment, and adequate management of patients.

Additionally, the combination of the RNA ISH and IHC on the same slide could provide further details about the specific localization of the virus in the morphological and cellular context.

Lesson learned from previous SARS-associated coronavirus guides us in the use of the in situ approach for the characterization of morphological features associated with SARS-CoV-2 infections in order to complete its etiopathogenetic landscape, still little known today.

Conclusively, the RT-PCR based on an extensive panel of targets, including E, N, S, and Orf1ab genes, represents a useful tool for the identification of SARS-CoV-2 on post-mortem FFPE lung samples from positive deceased patients as demonstrated in fresh samples. On the contrary, ISH is not a sensitive method to SARS-CoV-2 detection in post-mortem samples with a low viral load; however, the ISH approach could improve the knowledge on the localization of the virus in the cellular context, since this technique preserves the morphology.

## Supplementary information

ESM 1 (DOCX 16 kb)
