# Supplementary material for: Variable levels of spike and ORF1ab RNA in post-mortem lung samples of SARS-CoV-2-positive subjects: comparison between ISH and RT-PCR
Source: Virchows Arch. 2022 Feb 1;480(3):597–607. doi: 10.1007/s00428-021-03262-8 (PMC8805427; doi:10.1007/s00428-021-03262-8)
Supplement: Supplementary file 1 — (DOCX 16 kb) [file 428_2021_3262_MOESM1_ESM.docx]

| **CASE** | **AGE**  **(years)** | **GENDER** | **COMORBIDITIES** | **POST-MORTEM INTERVAL**  **(days)** | **DIAGNOSIS-DEATH INTERVAL**  **(days)** |
| --- | --- | --- | --- | --- | --- |
| **1** | 56 | M | Chronic nephropathy | 4 | 65 |
| **2** | 72 | F | Hypertension, cardiac hypertrophy | 3 | 38 |
| **3** | 67 | M | Hypertension | 4 | 40 |
| **4** | 45 | F | Obesity, diabetes mellitus | 5 | 68 |
| **5** | 78 | F | Hypertension, cardiac hypertrophy | 3 | 25 |
| **6** | 64 | M | Obesity, hypertension,  diabetes mellitus | 4 | 20 |
| **7** | 76 | M | Hypertension, cardiac hypertrophy | 5 | 80 |
| **8** | 80 | F | Hypertension, cardiac hypertrophy | 5 | 52 |
| **9** | 48 | M | Obesity, diabetes mellitus | 4 | 102 |
| **10** | 79 | M | Hypertension, cardiac hypertrophy | 5 | 60 |
| **11** | 73 | F | Hypertension, cardiac hypertrophy | 4 | 67 |
| **12** | 71 | F | Obesity, hypertension,  cardiac hypertrophy | 3 | 31 |
| **13** | 69 | M | Hypertension | 5 | 95 |
| **14** | 74 | M | Hypertension | 4 | 62 |
| **15** | 72 | F | Hypertension | 3 | 58 |
| **16** | 52 | M | Obesity, hypertension,  diabetes mellitus | 5 | 25 |
| **17** | 69 | F | Hypertension | 5 | 45 |
| **18** | 71 | M | Hypertension, cardiac hypertrophy | 4 | 58 |
| **19** | 65 | M | Hypertension | 4 | 122 |
| **20** | 61 | F | Hypertension | 3 | 110 |
| **21** | 58 | F | Chronic nephropathy | 5 | 54 |
| **22** | 79 | M | Hypertension | 4 | 87 |
| **23** | 64 | F | Obstructive chronic broncho-pneumopathy | 3 | 92 |
| **24** | 53 | F | Obstructive chronic broncho-pneumopathy | 5 | 46 |
| **25** | 48 | M | Obstructive chronic broncho-pneumopathy | 4 | 61 |
| **26** | 82 | M | Hypertension, cardiac hypertrophy | 4 | 52 |
| **27** | 77 | M | Hypertension, cardiac hypertrophy | 3 | 64 |

**Supp table 1: Clinical findings of the series.** M: males; F: females.
